# Remineralization Efficacy of Four Remineralizing Agents on Artificial Enamel Lesions: SEM-EDS Investigation

**DOI:** 10.3390/ma15134398

**Published:** 2022-06-22

**Authors:** Flavia Vitiello, Vincenzo Tosco, Riccardo Monterubbianesi, Giulia Orilisi, Maria Laura Gatto, Scilla Sparabombe, Lucia Memé, Paolo Mengucci, Angelo Putignano, Giovanna Orsini

**Affiliations:** 1 Department of Clinical Sciences and Stomatology (DISCO), Polytechnic University of Marche, 60126 Ancona, Italy; f.vitiello@pm.univpm.it (F.V.); v.tosco@pm.univpm.it (V.T.); r.monterubbianesi@staff.univpm.it (R.M.); g.orilisi@pm.univpm.it (G.O.); s.sparabombe@univpm.it (S.S.); l.meme@univpm.it (L.M.); a.putignano@univpm.it (A.P.); 2 Department of Materials, Environmental Sciences and Urban Planning (SIMAU) & UdR INSTM, Polytechnic University of Marche, 60131 Ancona, Italy; m.l.gatto@pm.univpm.it (M.L.G.); p.mengucci@univpm.it (P.M.)

**Keywords:** enamel, dental caries, remineralizing agents, CPP-ACP, fluoride varnish, nano-hydroxyapatite, SEM-EDS

## Abstract

Dental remineralization represents the process of depositing calcium and phosphate ions into crystal voids in demineralized enamel, producing net mineral gain and preventing early enamel lesions progression. The aim of the present study was to qualitatively and quantitatively compare the remineralizing effectiveness of four commercially available agents on enamel artificial lesions using Scanning Electron Microscopy (SEM) combined with Energy Dispersive Spectroscopy (EDS) techniques. Thirty-six extracted third molars were collected and randomly assigned to six groups (*n* = 6), five of which were suspended in demineralizing solution for 72 h to create enamel artificial lesions, and one serving as control: G1, treated with a mousse of casein phosphopeptide and amorphous calcium–phosphate (CPP-ACP); G2, treated with a gel containing nano-hydroxyapatite; G3, treated with a 5% SF varnish; G4, treated with a toothpaste containing ACP functionalized with fluoride and carbonate-coated with citrate; G5, not-treated artificial enamel lesions; G6, not demineralized and not treated sound enamel. G1–G4 were subjected to pH cycling over a period of seven days. Analyses of the specimens’ enamel surfaces morphology were performed by SEM and EDS. Data were statistically analyzed for multiple group comparison by one-way ANOVA/Tukey’s test (*p* < 0.05). The results show that the Ca/P ratio of the G5 (2.00 ± 0.07) was statistically different (*p* < 0.05) from G1 (1.73 ± 0.05), G2 (1.76 ± 0.01), G3 (1.88 ± 0.06) and G6 (1.74 ± 0.04), while there were no differences (*p* > 0.05) between G1, G2 and G6 and between G4 (2.01 ± 0.06) and G5. We concluded that G1 and G2 showed better surface remineralization than G3 and G4, after 7 days of treatment.

## 1. Introduction

Dental caries is the most prevalent chronic disease in children and adults worldwide, remaining the principal challenge in dentistry [1]. Dental caries is a biofilm-mediated, diet modulated, multifactorial, non-communicable, dynamic disease resulting in net mineral loss of dental hard tissues [2]. The modern concept of caries formation is based on the recurrence of various cycles of demineralization and remineralization phases, inducted by acid-producing bacteria in the oral micro-environment [3,4].

Indeed, the bacterial metabolism of fermentable carbohydrates, producing acids, causes the dissolution of mineral ions such as calcium and phosphate, which make up enamel hydroxyapatite crystals [5]. Thus, enamel demineralization can expose the dentine to the dentinal hypersensitivity related to the movement of fluids in dentinal tubules and the onset of caries disease [6]. However, the demineralized enamel prism possibly regenerates to its primordial stage if it is exposed to the oral conditions that promote remineralization [7].

Moreover, tooth decay is highly preventable and reversible at an early state, and the halt of enamel and dentin demineralization is possible with the inhibition of biofilm formation [8] and salivary protective factors [9]. In recent years, the focus in caries research has shifted to the development of methodologies for the non-invasive management of early caries lesions through remineralization to preserve tooth structure [10]. Indeed, remineralization can occur naturally, through the saliva buffering system, or biologically, induced by remineralizing agents [11].

Therefore, the scientific literature confirms that demineralization can be counteracted or reversed through several remineralization agents in non-cavitated carious lesions [6].

Several types and concentrations of remineralizing agents containing fluoride, calcium and phosphate ions are commercially available [12]. These agents release active ions which bond stably to the crystalline enamel structures, giving rise to newly formed crystals and reconstructing the damaged ones.

Fluoride ions represent the major mechanism in preventing enamel demineralization due to the formation of fluorapatite in enamel in the presence of calcium and phosphate ions produced during enamel demineralization by plaque bacterial organic acids [13,14,15]. Fluoride solutions come in low concentrations for daily use, such as toothpastes and mouthwashes, and higher concentrations for professional application, such as gels and varnishes [16].

Moreover, other compounds containing calcium and phosphate have been introduced, such as casein phospho-peptide and amorphous calcium–phosphate (CPP-ACP) and biomimicry hydroxyapatite, all of which have shown promising results.

CPP-ACP, a milk protein derivative, was commercially introduced since it has been proven to have anticariogenic effects [17]. CPP stabilizes high concentrations of calcium and phosphate ions with fluoride ions at the tooth surface by binding to pellicle and plaque [18]. ACP applies calcium ions (e.g., calcium sulphate) and phosphate ions (e.g., ammonium phosphate, sometimes in the presence of fluoride ions) separately so that ACP or amorphous calcium fluoride phosphate (F-ACP) forms intra-orally [15]. Evidence shows that the application of CPP-ACP may prevent demineralization and enhance remineralization through calcium and phosphate ion activities, thereby stabilizing ACP within the dental plaque in neutral or alkaline pH conditions and maintaining a state of supersaturation with respect to the enamel [19,20].

Another important source of mineral ions for the remineralization is nano-hydroxyapatite (N-HA), which presents crystals ranging in size between 50 and 1000 nm. N-HA was found to possess similar properties as a biological apatite [21]. The N-HA has a characteristic bond ability attributable to the size of nanoparticles, which considerably increases the bonding surface area, allowing available calcium or phosphate ions to bind to the enamel surface and filling the porosities of carious lesions [22,23]. N-HA crystals penetrate enamel pores and act as a template in the precipitation process, promoting crystal integrity and growth [24].

Nowadays, there is a gap in the literature regarding the short-term effect of these agents [25,26]. Therefore, we decided to analyze these products’ effects after 7 days of treatment as directed by the manufacturer of the tested agents.

There are still doubts and inaccuracies about their behavior, time and method of application. Despite the plethora of remineralizing agents, it is important to study new strategies and analyze different agents to improve the remineralization process.

The enamel hydroxyapatite crystals’ morphology and surface chemical composition are analytical indicators of dental tissue’s state of health. In particular, Scanning Electron Microscopy (SEM) is used to obtain high-resolution images, which give qualitative information on the enamel prism morphology of the samples [27], and Energy Dispersive Spectroscopy (EDS) allows us to quantify the main enamel components, such as calcium (Ca) and phosphorus (P), and their stoichiometric ratio [28,29].

Thus, in the present study, we aimed to analyze and compare the remineralizing effectiveness of four different commercially available agents on artificial human enamel lesions after seven days of treatment, by means of SEM combined with EDS.

## 2. Materials and Methods

### 2.1. Specimen Preparation

A total of 36 healthy human third molars were used. These teeth were extracted for orthodontic and periodontal reasons from the Department of Clinical Sciences and Stomatology of Polytechnic University of Marche. All patients were informed in advance that their extracted teeth would be used for scientific purposes, and written consent was obtained before the experiment. The collected teeth were screened for the presence of cracks, hypoplasia or white spot lesions and were excluded for having one of these. In selected teeth, remaining soft tissues, debris and stains were removed with hand-scaling instruments and stored in 0.5% *w/w* chloramine solution (NH_2_Cl) at room temperature. One single operator performed all procedures to avoid operator bias. Afterwards, samples were randomly allocated (*n* = 6) into six equal groups:

(1) **G1** (**Mousse Group):** demineralized as explained in Section 2.2 and then treated using GC Tooth Mousse (Recaldent GC, Europe), a mousse of CPP-ACP.
(2) **G2 (Nano-Hydroxyapatite Group):** demineralized as in Section 2.2, and then treated with Biorepair Desensitizing Enamel Repairer (Coswell oral care professional Spa, Italy), a gel containing zinc–hydroxyapatite.
(3) **G3 (Duraphat Group):** demineralized as in Section 2.2, and then treated with Duraphat (Colgate-Palmolive, USA), a varnish containing sodium fluoride (SF) 5%.
(4) **G4 (Biosmalto Group):** demineralized as in Section 2.2, and then treated with Biosmalto (Curasept Spa, Italy), a mousse containing F-ACP.
(5) **G5:** group demineralized as in Section 2.2, and then stored in artificial saliva (Biotene Oralbalance Gel, GSK, England).
(6) **G6:** group without any treatment, stored in artificial saliva (Biotene Oralbalance Gel, GSK, England).

The precise compositions of materials used in this study are listed in Table 1.

**Figure 1:**
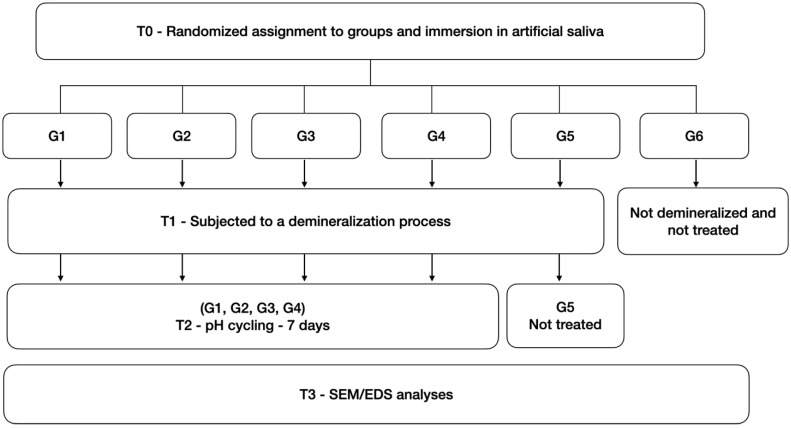
Schematic representation of experimental study design. G1 (Mousse Group), G2 (Nano-Hydroxyapatite Group), G3 (Duraphat Group), G4 (Biosmalto Group), G5 (group treated with the demineralizing solution), G6 (group without any treatment).

A schematic representation of this study design is presented in Figure 1. The outline represents significant steps in the experiment process.

### 2.2. Artificial Enamel Lesion Production

All groups, except G6, were immersed for 72 h in a demineralizing solution [30], containing 0.1 M lactic acid adjusted to pH 4.4 [31], in order to create an artificial initial lesion on the enamel surface [30]. After lesion formation, samples were thoroughly washed with deionized water and dried. For the entire period of the study, to simulate the oral cavity condition, after the demineralizing process, G5 was left in artificial saliva (Biotène Oral balance, GSK, England) composed of purified water, hydrogenated hydrolyzed starch, xylitol, hydroxyethylcellulose, polymetrhacrylate, beta-d-glucose, lactoperoxidase (12,000 units), lysozyme (12 mg), lactoferrin (12 mg), glucose oxidase (12,000 units), potassium thiocyanate and aloe vera, without any remineralizing treatment.

### 2.3. pH Cycling Condition

The pH cycle (demineralization–remineralization) was used to simulate pH fluctuation patterns in the oral cavity [30,32]: a demineralization cycle with 0.1 M lactic acid (adjusted to pH 4.4) for 6 h (30 mL for each sample) was performed, and secondly, a remineralization cycle with the agent’s application (2 min) in a thin layer using a microbrush on enamel surfaces was executed according to the manufacturer’s instructions.

The tested remineralizing agents’ pH levels were as follows: G1 (pH 6.7), G2 (pH 8.0), G3 (pH 5.7), G4 (pH 7.1).

The pH cycle was repeated once a day for seven days and samples were stored in solution in artificial saliva.

Finally, after the treatment procedures, all samples were carefully cleaned and dehydrated for evaluation using SEM-EDS.

### 2.4. SEM and EDS Analyses

Samples were air-dried, mounted on aluminum stubs and then observed by a TESCAN VEGA 3 LMU scanning electron microscope (Centre for Electron Microscopy—(CISMIN) Department of SIMAU, Polytechnic University of Marche, Ancona, Italy). SEM images were acquired to investigate the morphology of enamel and to search for surface damage at different magnifications: 200×, 500× and 1000×.

The chemical surface characterization was performed by means of EDS using EDAX Element Microanalysis (AMETEK Gmbh, EDAX Business Unit, Weiterstadt, Germany). EDS analysis was carried out on 3 sample areas with the following operating parameters: working distance of 15 mm, acceleration voltage of 25 kV and 500× magnification. The degree of remineralization was assessed by measuring the amount of phosphorus (P) and calcium (Ca) and calculating their ratio (Ca/P) in treated specimens after immersion in the artificial saliva. Results are reported as average value (AV) and standard deviation (SD).

### 2.5. Statistical Analysis

The EDS results were analyzed using descriptive statistics, and statistical inferences between experimental groups were determined by one-way ANOVA (analysis of variance) followed by Tukey’s test, using the statistical software Prism8 (GraphPad Software, San Diego, CA, USA). The group size was set to *n* = 6 for all experimental groups and significance was *p* < 0.05.

## 3. Results

**Figure 2:**
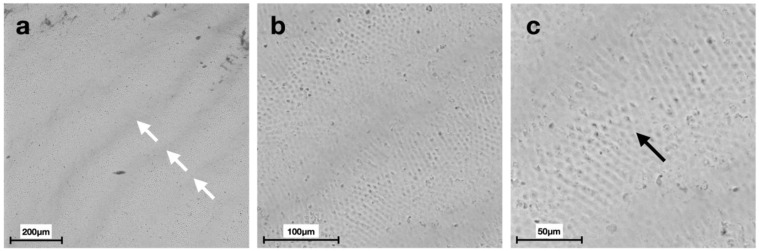
Scanning electron micrographs of G6 (group without any treatment): (**a**) the presence of perikymatas on the enamel surface (white arrows) at 200× magnification; (**b**,**c**) the crystalline structure composed of enamel prisms and the intact interprismatic area (black arrow) at 500× and 1000× magnification, respectively.

**Figure 3:**
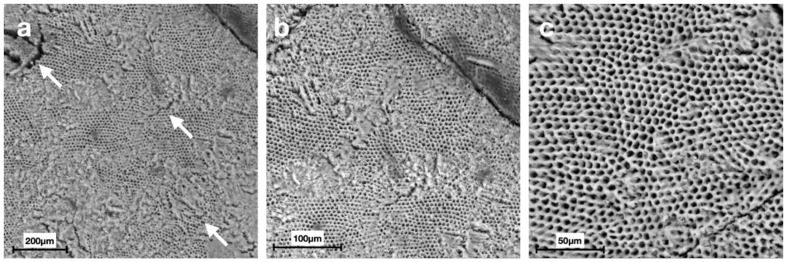
Scanning electron micrographs of G5 (group treated with the demineralizing solution): (**a**) an irregular surface with the presence of cracks and an early stage of destruction of the interprismatic substance (white arrows) at 200× magnification; (**b**,**c**) the “honeycomb structure” characterized by the selective dissolution of the apatite crystals inside the prisms, typical of the enamel demineralization morphology, at 500× and 1000× magnification, respectively.

**Figure 4:**
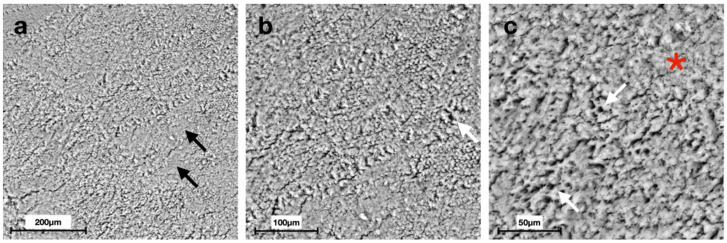
Scanning electron micrographs of G1 (Mousse Group): (**a**) intact morphology of the enamel surface (black arrows) at 200× magnification; (**b**,**c**) the partial recovery of the prism crystals (red asterisks) with the presence of a slight interprismatic dissolution (white arrows) at 500× and 1000× magnification, respectively.

**Figure 5:**
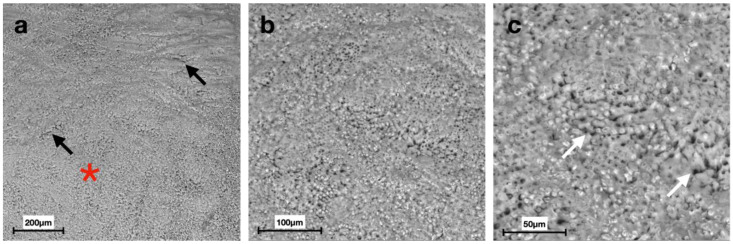
Scanning electron micrographs of G2 (Nanohydroxyapatite group): (**a**) a non-homogeneous surface with intact interprismatic areas (red asterisk) alternated by cracks in the enamel (black arrows) at 200× magnification; (**b**,**c**) the presence of enamel prisms with partially intact crystals with slight dissolution of the interprismatic area (white arrows) at 500× and 1000× magnification, respectively.

**Figure 6:**
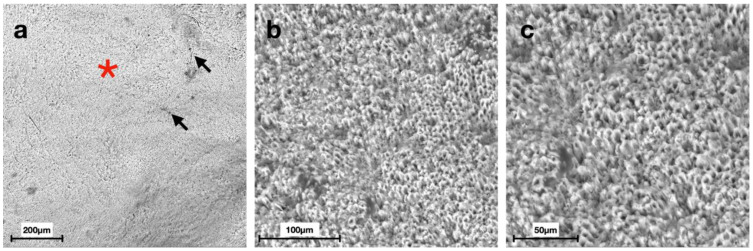
Scanning electron micrographs of G3 (Duraphat Group): (**a**) an irregular surface (red asterisk) with residues of the material evident on the surface (black arrows) at 200× magnification; (**b**,**c**) the presence of the enamel prisms with partially intact crystals and an almost restored structure of the interprismatic area at 500× and 1000× magnification, respectively.

**Figure 7:**
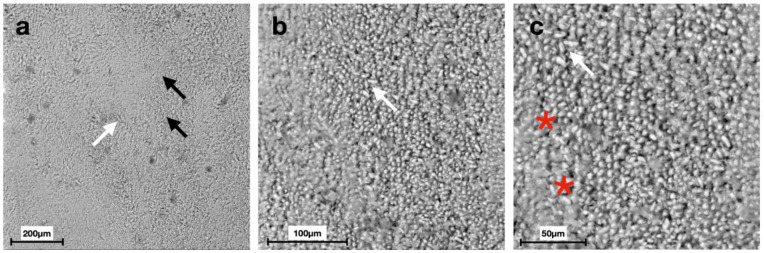
Scanning electron micrographs of G4 (Biosmalto Group): (**a**) a slight irregular surface of the enamel (black arrows), showing material residues on the surface (white arrow) at 200× magnification; (**b**,**c**) the irregular surface with a slight interprismatic dissolution (red asterisks) at 500× and 1000× magnification, respectively.

Scanning electron micrographs at different magnifications displayed distinct morphologies of the enamel surface for each group (Figure 2, Figure 3, Figure 4, Figure 5, Figure 6 and Figure 7).

SEM images of G6 displayed the typical regular aspect of the intact enamel crystalline structure (Figure 2). In G5, the integrity of the enamel prisms was severely affected, displaying an irregular surface with an early stage of the destruction of the interprismatic substance (Figure 3). G1 and G2 micrographs showed the partial recovery of the enamel prism crystals with the presence of a slight interprismatic dissolution (Figure 4 and Figure 5). G3 and G4 SEM micrographs presented an irregular enamel surface with residues of the material evident on the surface (Figure 6 and Figure 7).

**Figure 8:**
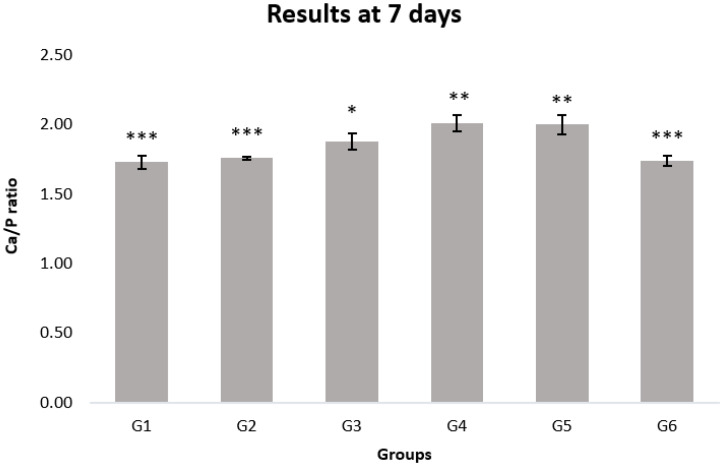
Ca/P ratio of all groups determined using EDS. One-way ANOVA with Tukey’s multiple comparison test; *p* < 0.05 is significant. Results are reported as average value and standard deviation. The following levels of statistical significance were considered: *p* < 0.05 *, *p* < 0.01 **, *p* < 0.001 ***. G1 (Mousse Group), G2 (Nano-Hydroxyapatite Group), G3 (Duraphat Group), G4 (Biosmalto Group), G5 (group treated with the demineralizing solution), G6 (group without any treatment).

The results of the statistical analysis of the Ca/P ratio assessed using EDS analysis are shown in Figure 8. The Ca/P ratio (AV ± SD) of the G5 (2.00 ± 0.07) is statistically different from G1 (1.73 ± 0.05), G2 (1.76 ± 0.01), G3 (1.88 ± 0.06) and G6 (1.74 ± 0.04) (*p* < 0.05). Contrarily, there are no significant differences between G6 and G1 and G2 (*p* > 0.05). Furthermore, G4 (2.01 ± 0.06) showed no statistically significant differences from G5 (*p* > 0.05).

## 4. Discussion

The era of preventive and minimally invasive dentistry dictates the need to develop new approaches to remineralize initial enamel lesions. Nowadays, many products, containing calcium, phosphate and fluoride in their bioavailable forms, are widely used to remineralize enamel structure and prevent dentin hypersensitivity in the form of toothpastes, mouth rinsing and gels [33]. In this in vitro study, the efficacy of four different agents on the enamel surface, after exposure to the acidic environment, was evaluated and compared.

The results demonstrated that by treating the demineralized enamel surface, the remineralizing agents provided ions that favored the subsurface mineral gain in different ways.

Compared to other studies in the literature [20,24,34], it is noteworthy that this experimental study is the first to analyze four different active ingredients commonly used in remineralizing agents (CPP-ACP, N-HA, SF, F-ACP) in order to assess the remineralization process of initial enamel lesions.

We used the SEM-EDS combined analytical technique for the qualitative investigation of the enamel surface morphology and the quantification of the Ca/P ratio as indications of enamel health [24]. Therefore, crystalline structure integrity and Ca/P values are important indicators of the agents’ effects on the enamel remineralization of several samples in the tested groups [35,36].

The stoichiometric form of hydroxyapatite is shown as Ca_10_(PO_4_)_6_(OH)_2_, and its Ca/P ratio is 1.67 [37], similar to that of G6 (1.74 ± 0.04), which is sound, not demineralized and untreated enamel. Indeed, as a result of the demineralization process, the amount of P decreases, as also found by Aoba et al. [38], thus resulting in the increase in the Ca/P ratio of G5 demineralized enamel (2.00 ± 0.07). Then, according to our results, the Ca/P ratio is inversely related to the health condition of the enamel. This result could probably be attributed to the action of lactic acid, used to perform demineralization, which acts on the phosphate component, increasing the ratio value.

SEM results of the demineralized enamel in G5 show an irregular morphology of the surface (Figure 3), defined as a “honeycomb structure”, with voids and numerous micropores due to the complete loss of prismatic structure integrity, which is preserved, instead, in G6 images (Figure 2) [39].

The four agents tested were compared with the Ca/P ratio values of sound enamel in G6. The closest values to G6 mean that the remineralizing treatment achieves a good percentage of enamel mineralization recovery.

Based on our results, among the tested products (G1–G4), CPP-ACP (G1) and N-HA (G2) are able to remineralize the enamel surface in a short time (7 days), returning to values very close to those of sound enamel (G6). SF (G3) differs slightly from G6, while F-ACP (G4) shows results that are similar to those of demineralized enamel (G5) in terms of the Ca/P ratio.

After seven days of treatment, SEM observations of G1 (Figure 4), treated with CPP-ACP, and G2 (Figure 5), treated with synthetic N-HA, highlight an almost complete remineralization of the enamel structure similar to the smooth sound enamel, as shown in Figure 2. Furthermore, regarding the EDS chemical analysis of G1 and G2, Ca/P ratios increasingly approached the G6 value that has not undergone demineralization, thus showing no significant differences between these three groups. In agreement with Yu et al. [40], our results suggest that the Tooth Mousse (G1) has a considerable effect on increasing the remineralization of enamel. In a study conducted by Li et al., CPP-ACP was able to remineralize primary lesions in contrast to placebo; however, contrary to our results, its effects are not significant compared to fluoride [41].

The specimens treated with N-HA(G2) exhibited a surface Ca/P ratio close to that of the biological enamel (G6) in comparison with SF and F-ACP, according to other studies [42,43,44]. In addition, in vitro dynamic pH cycling experiments have shown that N-HA has the potential to remineralize initial enamel lesions with a comparable or even superior efficacy to that of fluoride [45,46].

Since the cariostatic effects of fluoride have been discovered, caries prevention has largely relied on the ability of fluoride ions to inhibit enamel dissolution and enhance remineralization of incipient lesions [47]. Gao et al. showed that the use of a 5% SF varnish can remineralize incipient caries lesions, thus making this option an important method to inhibit enamel demineralization [48]. G3, treated with a varnish containing 5% of SF, showed a slight remineralization, but the Ca/P ratio (1.88 ± 0.06) remains slightly higher than that of G6 (1.74 ± 0.04) and presents statistical difference from all other groups. As confirmed by our study at seven days of treatment, 5% SF is less effective than other remineralizing products tested [49]. In contrast to our results, some studies state that 5% SF represents the maximum effective agent for remineralization [50,51].

It has been demonstrated that the F-ACP complex nanoparticles are able to attach to dental surfaces and to crystallize into hydroxyapatite in the oral environment, forming a new mineral phase contiguous to the biogenic one, and hence restoring the demineralized tissue into its native structure [52]. These findings are partially accepted in this study, since quantitative EDS results of G4 showed that Ca/P ratio (2.01 ± 0.06) does not present statistically different results compared to G5 (Figure 8), although scanning electron micrographs display an apparently partial intact structure of the enamel (Figure 7). Therefore, this treatment could benefit the partial restoration of the crystalline structure, while not affecting the amount of phosphorus. It is possible that the morphological shift of the surface precedes the compositional one (Ca/P). The capability of the F-ACP complex to remineralize the enamel surface is probably related to the time of the agent’s application.

One of the limitations of this research is the timing of the protocol study because after seven days, G4 was the only group that presented a non-statistical difference from G5. Therefore, the remineralization effectiveness of the tested agents applied for longer time is a stimulating concept emerging from our findings and will be taken as a starting point in future studies. In addition, we must consider the limits of the present in vitro study, as it is still far from simulating conditions present in the oral cavity. Nevertheless, the provided findings remain encouraging and, most importantly, demonstrate that the beneficial action of certain remineralizing products on dental enamel reorganization occurs at seven days.

## 5. Conclusions

Clinical significance: The initial enamel demineralization surface may be treated with topical use of remineralizing agents, achieving an almost complete remineralization of the surface and a reorganization of the prismatic structure of the enamel. In all groups tested, after seven days of remineralizing agents’ application, complete remineralization was not obtained, but rather a reorganization of the enamel structure from both quantitative and qualitative points of view. CPP-ACP (G1) and N-HA (G2) gave better results compared to SF (G3) and F-ACP (G4).

## Figures and Tables

**Table 1:** Chemical composition of the tested materials.

Material	Manufacturer	Ingredients
GC Tooth Mousse (G1)	Recaldent Europe	Pure water, glycerol, casein phosphopeptide–amorphous calcium–phosphate, D-sorbitol, sodium carboxymethyl cellulose, propylene glycol, silicon dioxide, titanium dioxide, xylitol, phosphoric acid, flavoring, zinc oxide, sodium saccharin, ethyl p-hydroxybenzoate, magnesium oxide, guar agam, propyl p-hydroxybenzoate, butyl p-hydroxybenzoate.
Biorepair Desensitizing Enamel Repairer (G2)	Coswell Spa, Italy	Aqua, zinc hydroxyapatite, hydrated silica, silica, sodium myristoyl sarcosinate, sodium methyl cocoyl taurate, sodium bicarbonate, aroma, sodium saccharin, phenoxyethanol, benzyl alcohol, sodium benzoate, citric acid, menthol.
Duraphat varnish (G3)	Colgate-Palmolive	Sodium fluoride, ethanol, white wax (E901), shellac (E904), rosin, putty, saccharin (E954), raspberry essence (containing ethyl butyrate, geraniol, iris resinoid, isoamyl acetate, absolute jasmine, vanillin and propylene glycol).
Biosmalto (G4)	Curasept Spa, Italy	Glycerin, PEG-8, silica, strontium acetate, calcium phosphate carbonate citrate fluoride, hydroxypropylcellulose, xylitol, acrylates/C10-30 alkyl acrylate crosspolymer, PEG-40 hydrogenated castor oil, sodium hyaluronate, potassium acesulfame, p-anisic acid, aroma, sodium hydroxide.

## Data Availability

The data presented in this study are available on request from the corresponding author.
